# Innovations in Airway Education: 3D Printed Neonatal and Pediatric Needle Cricothyrotomy Trainers

**DOI:** 10.21980/J8R928

**Published:** 2020-04-15

**Authors:** Zachary Hampton, Alex Davis, Andrew Kalnow

**Affiliations:** * OhioHealth Doctors Hospital, Department of Emergency Medicine, Columbus, OH

## Abstract

**Audience:**

Three dimensional printed cricothyrotomy trainers are designed to be used at all levels of training in emergency medicine, both for skill acquisition and to prevent skill atrophy.

**Introduction:**

Simulation has long provided a means to train providers on rarely performed procedures.^1^,^2^ For example, surgical cricothyrotomy has commonly been practiced in a simulated environment almost exclusively via low-fidelity models.^3^–^6^ What seems to be lacking in this training is simulation of needle cricothyrotomy for the pediatric population. Needle cricothyrotomy remains the standard of care for temporary airway management in a “can’t intubate, can’t oxygenate” scenario for pediatric patients. In order to address this educational deficiency, we designed a 3D printed pediatric needle cricothyrotomy trainer using evidenced-based cadaveric literature demonstrating neonatal cricothyroid membrane dimensions. These trainers will serve as a simulation tool that will help educate emergency medicine residents to perform a rare, high-stakes procedure within a controlled environment.

**Educational Objectives:**

By the end of this educational session, participants should be able to:

1. Discuss indications and contraindications for needle cricothyrotomy in the pediatric population.
2. Assemble the equipment needed to complete a needle cricothyrotomy.
3. Describe and perform the steps of neonatal and pediatric needle cricothyrotomy.
4. Discuss post-procedure ventilation options.

**Educational Methods:**

Trainers were created to enhance needle cricothyrotomy training in this rarely performed, yet critical procedure. It was felt that this procedure was only discussed in concept. There was no hands-on, procedure-based option available to realistically reproduce the procedure in a controlled training environment, and we felt the creation of a task trainer was ideal to address this deficiency. Once the trainers were created, we curated assigned pre-reading using a flipped classroom approach. The idea was to provide learners with multiple resources including textbook chapters, blogs, and podcasts, so that they could choose one or two resources that matched their learning style. We felt this was the best way to ensure learner retention. Additionally, we created a PowerPoint presentation to illustrate the stepwise procedure, required equipment, indications/contraindications, and ventilation aspect of the procedure, which can be reviewed with learners prior to performing the hands-on portion of the skills station.

In order to create such a trainer, we worked in conjunction with the OhioHealth Simulation Department to obtain a 3D printer. Adult cricothyrotomy trainers were previously purchased from The Airway App, a company specializing in airway management education. The company also provides a standard template library (.STL) file that contains code for 3D printing cricothyrotomy trainers. Using this code, we made changes to the scale in order to create a smaller model that would resemble neonatal, school-aged, and adolescent patients (25%, 33%, and 50% of the original adult trainer, respectively). These scale models, particularly the neonatal model, were chosen based on the neonatal cadaveric measurements researched by Navasa, et al.^7^

To prepare the trainer for use, sim skin and sim tape were used to cover and obscure the landmarks. Learners were given the materials previously discussed in preparation of the skills lab. Although all sizes of trainers were used by the learners, particularly attention was paid to the neonatal trainer. Emphasis was placed on the difficulty finding landmarks during this procedure. Instructors were available for the entirety of the skill station in order to answer questions and give direct feedback. Debriefing was performed at the end of the skills session.

**Research Methods:**

Post-simulation surveys were completed by PGY 1–4 emergency medicine resident physicians assessing pre- and post-simulation procedural comfortability using a 5-point Likert scale. In this survey, 1 represented “not comfortable at all” while 5 represented “completely comfortable.” Two, three, and four showed a gradual increase in comfortability, respectively. Median pre- and post-scores were calculated with interquartile ranges (IQR). A two-tailed Mann-Whitney U test was performed for statistical significance. Realism and future use were also surveyed.

**Results:**

Thirty-one emergency medicine resident physicians ranging from PGY1 to PGY4 completed the post-simulation survey. Median pre-simulation score was 2 (IQR 1–2.5) and post-simulation score was 4 (IQR 3–4). The two-tailed Mann-Whitney U test showed statistical significance at p<0.01. Ninety-seven percent of participants felt the trainers were realistic and 100% would recommend them for future simulation use.

**Discussion:**

Novel 3D printed needle cricothyrotomy trainers are a much-needed addition to emergency medicine procedural training. Specifically, we focus on the use of low-cost, high-fidelity trainers for educating emergency medicine resident physicians. By using a pre-templated .STL file, we were able to manipulate sizes and dimensions to create several simulation trainers for simulating needle cricothyrotomy. Our results show that these trainers are valuable to emergency medicine procedural training, increasing resident comfortability. Furthermore, participants feel this is realistic and would recommend this for future simulations. Given the relative ease and low-cost nature of these trainers, in addition to their proven benefit with residents, we hope that others will be able to use similar trainers to help develop needle cricothyrotomy skills at all levels of training.

**Topics:**

Needle cricothyrotomy, pediatrics, difficult airway, 3D printing, education.

## USER GUIDE

[Table t1-jetem-5-2-i1]

**Table t1-jetem-5-2-i1:** 

List of Resources:	
Abstract	1
User Guide	4

**Learner Audience:**

Medical Students, Interns, Junior Residents, Senior Residents, Advanced Practice Providers, Attending Physicians

**Time Required for Implementation:**

Timing for implementation largely depends on 3D printer availability. Many universities and hospital systems already have printers available which can be used by outside parties. Once the printer and STL code is obtained, printing time depends on the size of the trainer. In this case, printing time ranged from 25 minutes to several hours depending on the size of the trainer. The basic setup for this simulation takes only a few minutes. This requires taping the printed needle cricothyrotomy trainers to a hard surface and covering them with sim tape, followed by sim skin. A more elaborate setup with accompanying head and body is possible; however, this would require a more involved setup which this session did not entail. We recommend approximately 30 minutes of allotted time for acquisition and skill development. If desired by the instructor, this time can include a brief review of the associated PowerPoint. Debrief time was also minimal, taking only approximately 5 minutes to discuss the difficulty of palpating landmarks and the incredibly small size of the larynges and cricothyroid membranes. Some residents also discussed difficulty with setting up the equipment and connecting all of the individual pieces together.

**Recommended Number of Learners per Instructor:**

One instructor can give a pre-simulation lecture to any number of participants. Two learners per trainer is optimal, though this is only a recommendation and can be expanded depending on available resources. The number of instructors required to implement the simulation largely depends on the comfortability and training level of the instructor(s). Only one instructor is needed to give the pre-procedure PowerPoint, though many may be used to float during the skill lab. During debrief, all learners can come together to discuss their experience with the trainers. In total, we recommend 30 minutes with approximately 5 minutes of pre-procedure PowerPoint review and 5 minutes for debrief.

**Topics:**

Needle cricothyrotomy, pediatrics, difficult airway, 3D printing, education.

**Objectives:**

By the end of this educational session, participants should be able to:

1. Discuss indications and contraindications for needle cricothyrotomy in the pediatric population.
2. Assemble the equipment needed to complete a needle cricothyrotomy.
3. Describe and perform the steps of neonatal and pediatric needle cricothyrotomy.
4. Discuss post-procedure ventilation options.

### Linked objectives and methods

Rare procedures such as needle cricothyrotomy require dedicated learning sessions and trainers to develop the knowledge and skill required for procedural success. This procedure training session allows learners to review the situations where this procedure may be indicated, as well as the equipment needed and procedural steps through an introductory PowerPoint and associated pre-simulation reading (objectives 1 and 4). Following the presentation, learners will then describe and perform the procedure on the 3D printed cricothyrotomy trainer, allowing application and tactile feedback through deliberate practice (objectives 2–4).

### Recommended pre-reading for instructor

Note - not all of these are required. We recommend picking 1–2 resources that you prefer from the list below.

1. Nagler J, Mick N. Airway management for the pediatric patient. In: Walls RM, Hockberger RS, Gausche-Hill M, et al, eds. *Rosen’s Emergency Medicine: Concepts and Clinical Practice.* 9th ed. Philadelphia, PA: Elsevier; 2018:1994–2004.
2. Herbert R, Thomas D. Cricothyrotomy and percutaneous translaryngeal ventilation. In: Roberts JR, Custalow CB, Thomsen TW, eds. *Roberts and Hedge’s Clinical Procedures in Emergency Medicine and Acute Care*. 7th ed. Philadelphia, PA: Elsevier; 2019: 127–141.
3. Hansen ML, Eriksson C. Intubation and ventilation of infants and children. In: Tintinalli JE, Ma O, Yealy DM, eds. *Tintinalli’s Emergency Medicine: A Comprehensive Study Guide.* 9^th^ ed. New York, NY: McGraw-Hill; 2020.
4. Mason J, Herbert M, Swadron S. Pediatric airway management - needle cricothyrotomy. EM: RAP. https://www.emrap.org/episode/c3pediatric2/c3pediatric4. Published September 2019. Accessed February 29, 2020.
5. Weingart S. Podcast 053 – Needle vs. knife: part I. EMCrit-RACC. https://emcrit.org/emcrit/cricothyrotomy-needle-or-knife/. Published August 9, 2011. Accessed February 29, 2020.

### Learner responsible content (LRC)

In addition to the resources cited above, learners might also find these resources helpful. We recommend allowing the learner to pick 1–2 below.

1. Ameli J. The cricothyrotomy part 3: pediatric Points. Brown Emergency Medicine-The Educational Blog of the Brown EM Residency. https://blogs.brown.edu/emergency-medicineresidency/the-cricothyrotomy-part-3-pediatric-points/. Published December 9, 2015. Accessed February 29, 2020.
2. McVea S. Cricothyroidotomy. Paediatric Emergencies. https://www.paediatricemergencies.com/intubationcourse/course-manual/cricothyroidotomy/. Published April 2018. Accessed March 1, 2020.
3. Needle cricothyroidotomy. UCLA Center for Prehospital Care. https://www.cpc.mednet.ucla.edu/sites/default/files/Refresher131112/Day1/Transition/NeedleCricothyroidotomy.pdf. Accessed March 1, 2020.

### Implementation Methods

The session should begin with a single pre-simulation lecture (see associated PowerPoint) lasting approximately 5–10 minutes. This will ensure all learners have a baseline understanding of the needle cricothyrotomy procedure, although all learners should have reviewed the associated material prior to the skills station. Indications and contraindications should be reviewed, and all relevant supplies gathered.

Learners are allowed to practice using all sizes of the trainer, with a particular emphasis on the neonatal trainer because this is most difficult procedurally. Trainers can be used indefinitely since they are not damaged during the simulation. Sim skin and foam tape will withstand several attempts on the trainer, but will need to be changed out eventually. This depends largely on the number of attempts each learner is taking.

We used a bag-valve for teaching post-procedure ventilation because we did not have access to a jet ventilation system. This option is, however, covered in the associated PowerPoint and accompanying material. If available, we would recommend practicing with the jet ventilation system. Ultimately, learners will be best served by using whatever is available at the location where they practice.

### List of items required to replicate this innovation

1. 3D printer (ie, Prusa MK3); https://www.prusa3d.com/original-prusa-i3-mk3/
2. STL Airway App file: http://www.airwaycollaboration.org/3d-cric-trainer-1
3. Software to manipulate file
  - Blender – 3D modeling software: https://www.blender.org/.
  - Slic3er – used to lay out the model and upload the file into the printer: https://slic3r.org/.
4. Large SD card to support file size
5. Filament of choice (PLA was used for these trainers; at multiple retailers including Amazon and Best Buy)
6. Silicone skin (you do not need all of these; any one or two in combination will work)
  - Sim Skin: https://sim-skin.myshopify.com/.
  - Dragon Skin: https://www.reynoldsam.com/product/dragon-skin/.
  - Suture pad: https://www.amazon.com/Durable-3-Layer-Powermesh-Durability-Training/dp/B07F7LD1M4/ref=pd_sbs_328_img_2/139-5934544-0171968?_encoding=UTF8&pd_rd_i=B07F7LD1M4&pd_rd_r=36c8cd4f-3a58-4a6d-992df22ccd43103d&pd_rd_w=7lAI8&pd_rd_wg=hD0v6&pf_rd_p=5cfcfe89-300f-47d2-b1ada4e27203a02a&pf_rd_r=4F7Y5EJE2PVV0E4FR0W1&psc=1&refRID=4F7Y5EJE2PVV0E4FR0W1.
7. 3M surgical foam tape: https://www.3m.com/3M/en_US/company-us/all-3m-products/~/3M-Microfoam-Surgical-Tape/?N=5002385+3293321966&rt=rud.
8. Angiocatheter (various sizes ranging from 16g to 20g)
  - Ideally, these should have a Leur lock in order to connect flushes. Auto-retracting safety needles work but cannot be attached to 10 cc syringes until after the needle is withdrawn. This increases the risk of tracheal posterior wall perforation and esophageal injury.
9. 3 ml syringe
10. 10 cc saline flush

### Approximate cost of items to create this innovation

Prices range widely for 3D printers, from a couple hundred dollars to thousands. In this case, the Prusa MK3 costs $749 ($999 assembled).^8^ Polylactic acid (PLA) printer filament (the wound plastic used for printing) costs approximately $20 per kilogram, which can make many trainers. Software used for 3D printing will often estimate the cost per print. For our trainers, production cost is minimal. The smallest trainer (neonatal) costs merely $0.45, increasing up to a couple of dollars for the larger trainers. Various products can be used to obscure landmarks. We used a combination of foam surgical tape ($20–$30 depending on width and number of rolls) and silicone suture pad (approx. $30 for 5x7” pad), though many products exist and all essentially serve the same purpose. It is important to note that these supplies are bulk size and, once purchased, will be adequate for many sessions. Other supplies, including needle catheters, endotracheal tubes, flushes, syringes, and bag-valves were all obtained from the emergency department or through our simulation department.

### Detailed methods to construct this innovation

1. From The Airway App: http://www.airwaycollaboration.org/, download the .STL file containing the code for the adult sized cricothyrotomy trainer.[Fig f1-jetem-5-2-i1]
  **Figure f1-jetem-5-2-i1:**
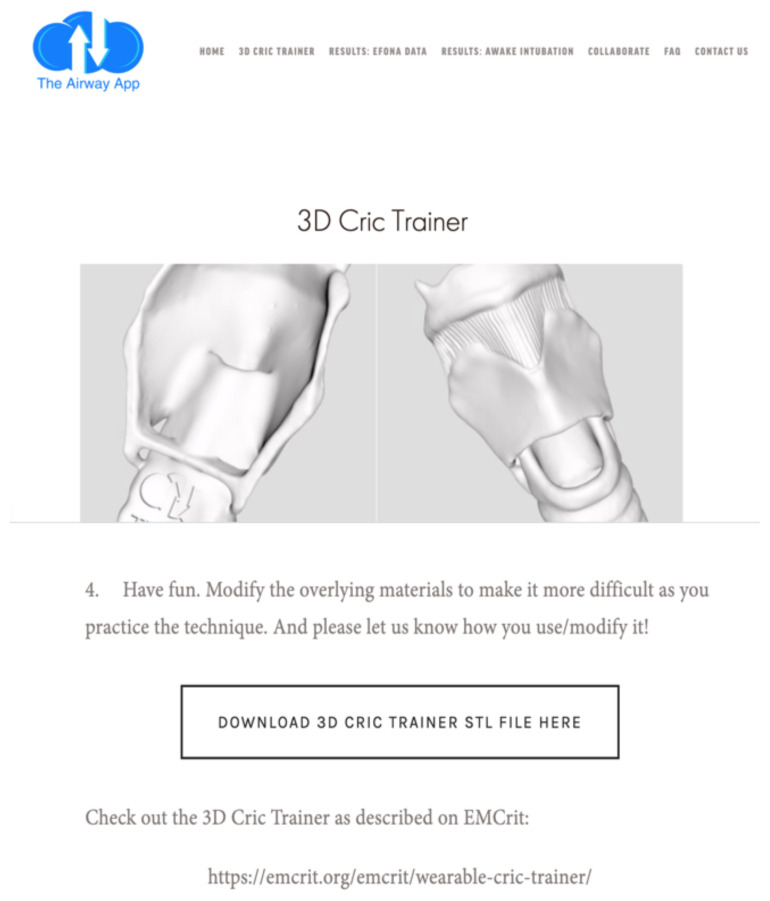
2. Use Blender (or other similar software) to manipulate the model if needed. Alternatively, the .STL file can be uploaded directly into Slic3r (or similar) and scaled, which is the process that we used. This may require a large SD card to hold and transfer the file.[Fig f2-jetem-5-2-i1]
  **Figure f2-jetem-5-2-i1:**
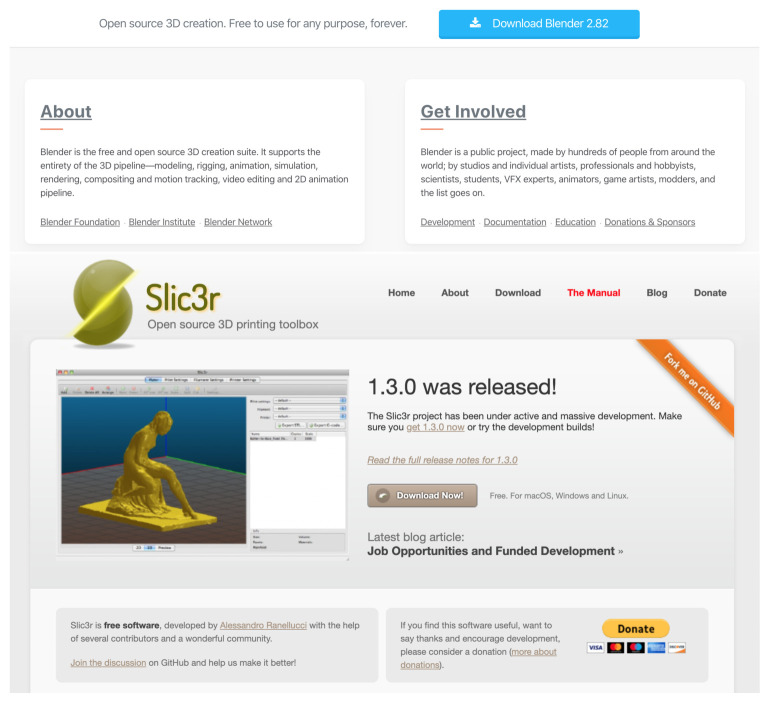
3. Load filament (PLA) into the printer. Program the 3D printer to run the model.
4. Various sizes can be printed as long as the material can withstand the changes from a structural standpoint. In this case, models were scaled to approximately 25%, 33%, and 50% of the adult trainer (in order from right 25% to left adult trainer, and top 25% to bottom 50%).[Fig f3-jetem-5-2-i1]
  **Figure f3-jetem-5-2-i1:**
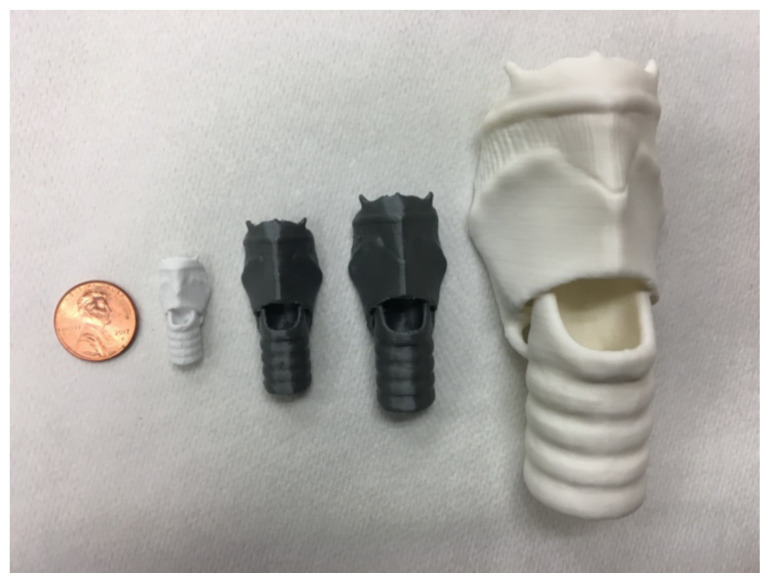
[Fig f4-jetem-5-2-i1]
  **Figure f4-jetem-5-2-i1:**
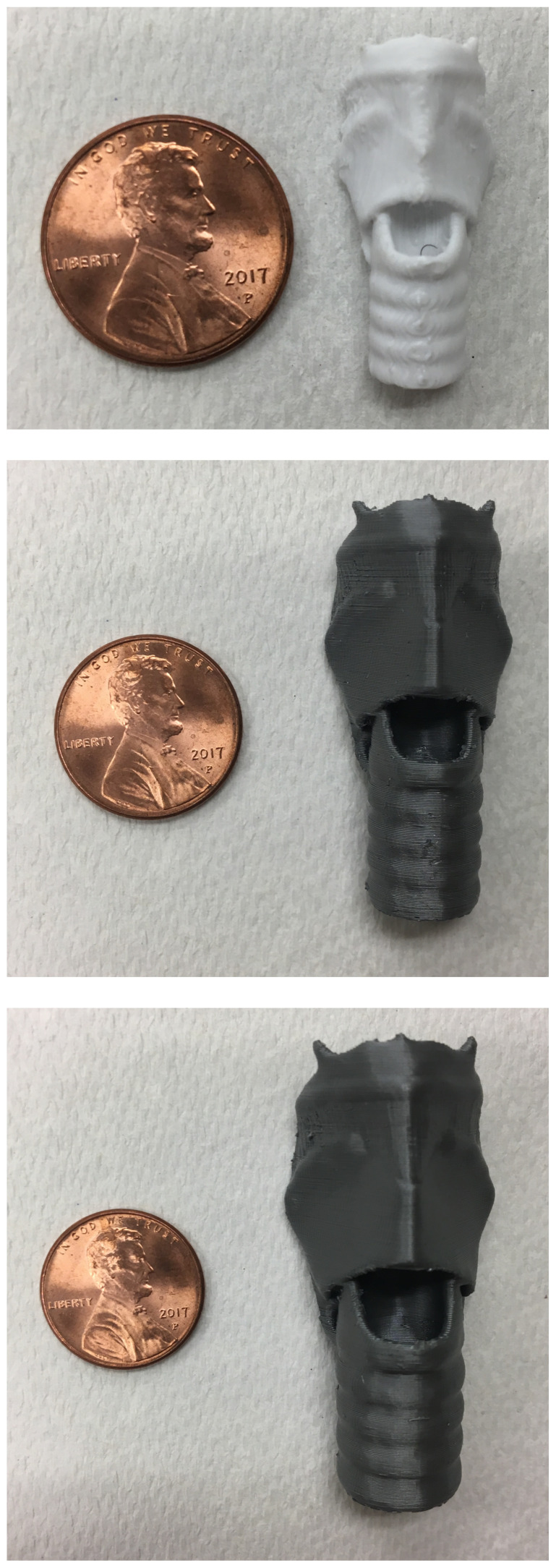
5. Once models are printed, cover with either Sim Skin and/or 3M surgical foam tape in order to obscure landmarks and increase realism.[Fig f5-jetem-5-2-i1]
  **Figure f5-jetem-5-2-i1:**
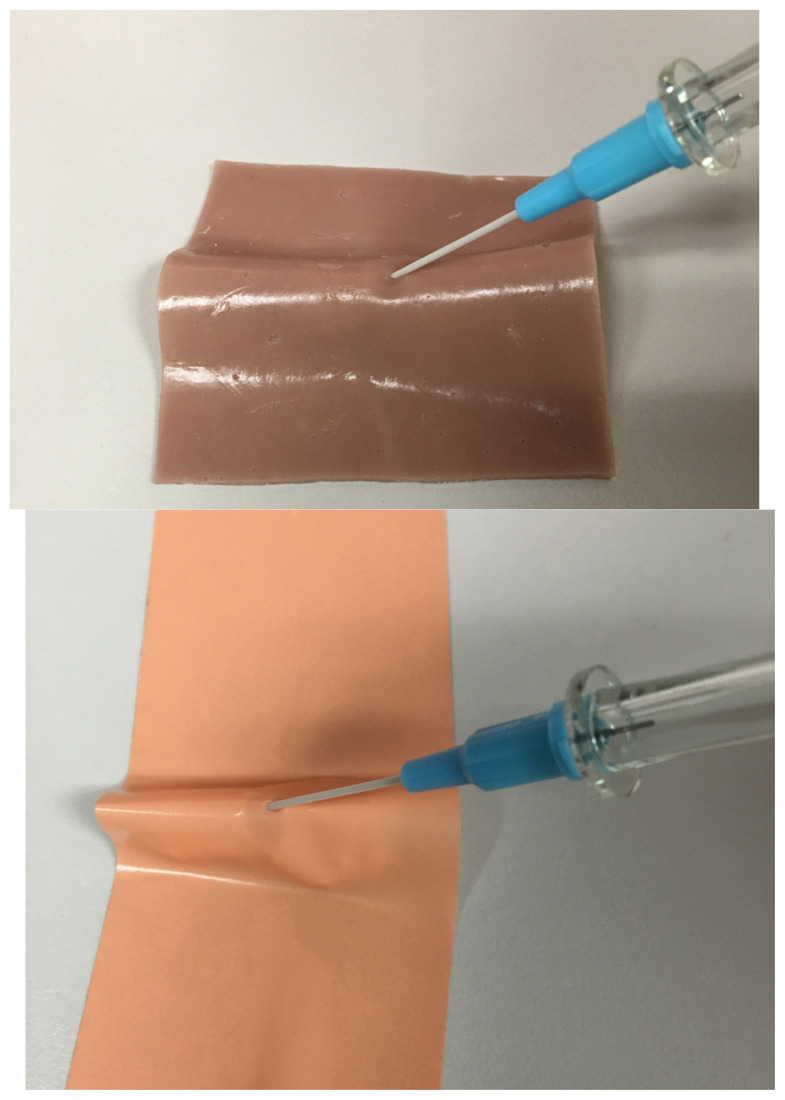
6. Simulation stations are created using trainers and supplies as listed above (bag-valve mask, endotracheal tubes, angiocatheter, 10 cc flush, 3 ml syringe).
7. Instructors give a 5–10-minute introduction lecture to review the procedure.
8. Learners should have 20–30 minutes to practice the procedure.

### Associated content

- See associated PowerPoint
[Fig f6-jetem-5-2-i1]
  **Figure f6-jetem-5-2-i1:**
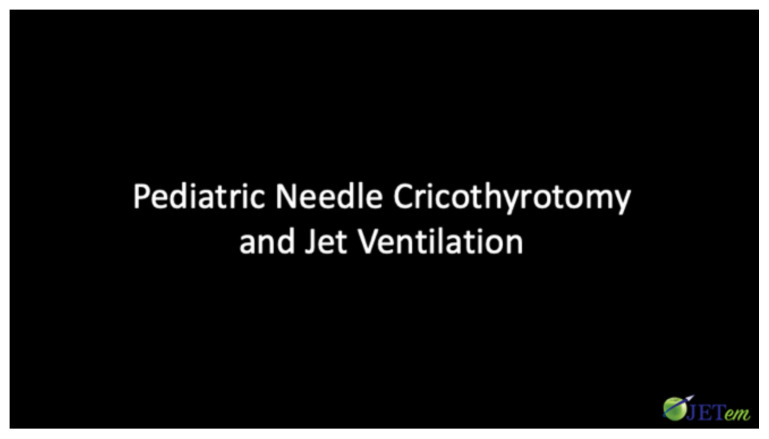
- Or, see the link for access for the PowerPoint that can be used during the educational sessions: https://drive.google.com/file/d/1yUg0YtT8swwcq7fBHmegEMIQj5FU2c_1/view?usp=sharing.

### Results and tips for successful implementation

Participants completed a post-simulation assessment survey (N=31) in which comfortability was measured on a 1–5 scale, with 5 being completely comfortable in performing the procedure and 1 being not comfortable at all. Median pre-simulation and post-simulation scores were calculated with interquartile ranges (IQR). Statistical analysis was performed using a two-tailed Mann-Whitney U test. Median pre-simulation score was 2 (IQR 1–2.5) and median post-simulation score was 4 (IQR 3–4). Results were statistically significant (p<0.01). Ninety-seven percent of participants felt that the simulation was realistic, and 100% of participants would recommend it for residents or attendings in the future. Our data confirms the efficacy of this low-cost, high fidelity trainer in resident simulation.

For a higher fidelity experience, the instructor can create a formal simulation where the participant is asked to assess a pediatric patient in respiratory distress, eventually leading to needle cricothyrotomy performed with the same trainers. Although no data was collected, the PGY 3 and 4 participants of our survey completed a full simulation as just described. Overall, comments were positive because residents appreciated performing the procedure as part of a larger simulation and pediatric resuscitation.

## Supplementary Information

Please see associated PowerPoint file
